# Ell3 functions as a critical decision maker at the crossroad between stem cell senescence and apoptosis

**DOI:** 10.1186/s13287-019-1137-9

**Published:** 2019-01-17

**Authors:** Jae-Yong Lee, Soo-Hong Lee, Kwang-Soo Kim, Keun-Hong Park, Kyung-Soon Park

**Affiliations:** Department of Biomedical Science, College of Life Science and CHA Stem Cell Institute, CHA University, 335, Pangyo-ro, Bundang-gu, Seongnam-si, Gyeonggi-do 463-400 Korea

**Keywords:** Ell3, p53, Bcl-2, Senescence, Somatic stem cell, Apoptosis

## Abstract

**Background:**

Ell3 is a RNA polymerase II elongation factor that has various cell type-dependent functions, such as regulating the differentiation efficiency of embryonic stem cells and sensitizing cancer cells to anticancer drugs. However, there has been little research on the role of Ell3 on the regulation of senescence and apoptosis of stem cells.

**Methods:**

We analyzed the senescence of Ell3-suppressed stem cells by mitochondrial activity, β-gal (+) cells, and lineage differentiation efficiency. The apoptosis of Ell3-overexpressing stem cells was analyzed by Annexin V staining, Immunoblot, and Live&dead assay. In addition, chromatin immunoprecipitation and luciferase assays were used to demonstrate p53 functions as a direct transcriptional activator of Ell3.

**Results:**

Suppression of Ell3 expression induced senescence in stem cells by increasing Bcl-2 expression. Unlike the effect of Ell3 suppression, the ectopic expression of Ell3 induces apoptosis of stem cells and induces apoptosis of adjacent cells. In addition, p53 functions as a direct transcriptional activator of Ell3 during the stem cell apoptosis.

**Conclusions:**

We suggest that the function of Ell3 is associated with the p53-Bcl2 axis in both senescent and apoptotic ADSCs.

**Electronic supplementary material:**

The online version of this article (10.1186/s13287-019-1137-9) contains supplementary material, which is available to authorized users.

## Background

Adult stem cells are successfully expanded in culture and will eventually undergo senescence at the end of their replicative lifespan in vitro [1]. Replicative senescence is characterized by senescence-associated features including a permanent state of growth arrest, morphological changes, and high expression levels of tumor suppressors such as p16^INK4a^, p21^Cip1^, and p53 [2, 3]. While the p16^INK4a^/RB pathways and p53/p21^Cip1^ are considered key regulators of cellular senescence [4], senescence can be induced independent of these pathways. For example, Raf-1 and BRAF induce senescence depending on factors other than p16 [5, 6]. The ability of the cell death inhibitor Bcl-2 to promote senescence in cells lacking p53 or p16 indicates that Bcl-2 also induces p53-independent senescence [7]. The senescence of cells is usually accompanied by robust changes in the secretion of senescence-associated secretory phenotype (SASP) factors [8]. SASP factors consist of distinct secreted proteins including inflammatory cytokines, growth factors, and proteases [9]. For this reason, senescence is implicated in chronic diseases of aging, such as macular degeneration and osteoarthritis [10, 11].

Although the isolation of mesenchymal stem cells (MSCs) according to current criteria produces heterogeneous, nonclonal cultures of stromal cells containing stem cells with different multipotential properties, committed progenitors, and differentiated cells, the promising features of these cells, including their regenerative properties and potential to differentiate into diverse cell lineages, make MSCs a promising candidate for regenerative medicine [12].

However, senescence of MSCs accompanied by an imbalance between osteoblast and adipocyte differentiation is one major hurdle in the clinical application of MSCs [13, 14]. While FOXP1 reportedly controls senescence and cell-fate commitment via interactions with key modulators of adipogenesis and osteogenesis in bone marrow MSCs [15], the molecular network orchestrating the senescence and reciprocal balance between adipo-osteogenic-MSC differentiation remains largely unknown.

Ell3 is a member of the eleven-nineteen lysine-rich leukemia (Ell) family of RNA polymerase II transcription elongation factors and is enriched in the testis. In embryonic stem cells, Ell3 plays an essential role in the activation of developmentally regulated genes by priming these genes for the recruitment of a proper transcription initiation complex during differentiation [16].

Recently, we reported that the treatment of mouse embryonic fibroblasts (MEFs) with conditioned media obtained from Ell3-suppressed adipose-derived stem cells (ADSCs) results in the induction of proinflammatory cytokine genes such as interleukin-1 and interleukin-6 [17]. As these cytokines belong to SASP factors and interleukin-6 reportedly induces the premature senescence of young cells [18, 19], whether Ell3 expression is associated with ADSC senescence and whether conditioned Ell3 media suppresses the ADSC-induced senescence of MEFs remain to be determined.

In this study, we studied the effect of endogenous Ell3 on the senescence and apoptosis of ADSCs. The suppression of Ell3 results in the senescence of stem cells characterized by growth arrest, enhancement of Bcl-2 expression accompanied by p53 suppression, mitochondrial dysfunction, and off-balance adipo-osteogenic differentiation. A senolytic drug targeting Bcl-2 rebalances the differentiation of Ell3-suppressed stem cells that are primed for adipogenesis. By contrast, ectopic Ell3 expression induces ADSC apoptosis as a result of enhanced p53 expression. We further revealed that p53 directly regulates Ell3 expression in adipose stem cells, which suggests the possibility of the reciprocal regulation of Ell3 and p53 to modulate stem cell senescence and apoptosis.

## Methods

### Cell culture

ADSCs (cat no. PCS-500-011) were purchased from ATCC (Manassas, VA, USA) and were cultured in α-MEM (cat no. 12571-071, Thermo Fisher Scientific, MA, USA) containing 10% fetal bovine serum (cat no. 12484-010, Thermo Fisher Scientific) and 1% penicillin/streptomycin (cat no. 15140148, Thermo Fisher Scientific). Bone marrow-derived stem cells (BM-MSCs) were purchased from Lonza Walkersville Inc. (cat no. PT-2501, Walkersville, MD, USA). After thawing, BM-MSCs were cultivated in MSCGM Basal Medium (cat no: PT-3001; Lonza Walkersville Inc.) in a humidified 5% CO2 incubator at 37 °C.

MCF7 cells were cultured in DMEM containing 10% fetal bovine serum and 1% penicillin/streptomycin. MCF10A cells were cultured in DMEM/F12 (cat no. 11320082 Thermo Fisher Scientific) containing 10% horse serum (cat no. #16050-122 Invitrogen, CA, USA), 20 ng/ml epidermal growth factor (EGF) (cat no. E9644 Sigma-Aldrich, St. Louis, MO, USA), 0.5 mg/ml hydrocortisone (cat no. H0888 Sigma-Aldrich), 100 ng/ml cholera toxin (cat no. c-8052 Sigma-Aldrich), 10 μg/ml insulin (cat no. I-1882 Sigma-Aldrich), and 1% penicillin/streptomycin.

### Antibodies and immunoblot analysis

The following antibodies were used in this study: anti-Ell3 (cat no. ab67415, Abcam, Cambridge, UK), anti-β-actin (cat no. sc-47778 Santa Cruz, CA, USA), anti-p53 (cat no. 2527, Cell Signaling, MA, USA), anti-acetyl p53 (cat no. 2525, Cell Signaling, MA, USA), anti-phospho p38 (cat no. 4511, Cell Signaling), anti-p38 (cat no. 8690, Cell Signaling), anti-caspase3 (cat no. 9662, Cell Signaling), anti-BAX (cat no. 5023, Cell Signaling), anti-p21 (cat no. sc-471, Santa Cruz, CA, USA), anti-p16 (cat no. sc-468, Santa Cruz, CA, USA), anti-pRb (cat no. 2181, Cell Signaling), and anti-BCL2 (cat no. 4223, Cell Signaling). For immunoblot analysis, cells were lysed in tissue lysis buffer (cat no. 9803, Cell Signaling, Denver, USA), and total cell extracts were resolved by sodium dodecyl sulfate-polyacrylamide gel electrophoresis (SDS–PAGE) and then transferred to immunoblot PVDF membranes (cat no. IPVH00010, Millipore, MA, USA). The membranes were blotted with antibodies, and immunoreactivity was detected by enhanced chemiluminescence (cat no. 34579, Thermo Fisher Scientific).

### siRNA-mediated suppression of Ell3

ADSCs were transfected with siEll3 (M-014601-01-0005), which was purchased from Dharmacon (distributed by Thermo Scientific/AbGen, Epsom, UK). Cells were transfected with either siRNA targeting Ell3 or nonspecific siRNA using Lipofectamine 3000 (Invitrogen) in OPTI-MEM (Invitrogen) according to the manufacturer’s instructions.

### Real-time reverse transcription PCR (qRT-PCR)

Total RNA was isolated using TRI-reagent (Sigma-Aldrich). In total, 1 μg of total RNA was reverse transcribed using the 1st Strand cDNA Synthesis kit (LeGene, San Diego, CA, USA) according to the manufacturer’s protocol. qRT-PCR was performed in triplicate using the primers listed in Additional file 1: Table S1 with TOPreal qPCR 2X PreMIX (Enzynomics, Daejeon, Korea) and the CFX96 Real-time System (Bio-Rad Laboratories, Richmond, VA, USA). Expression levels were normalized to that of glyceraldehyde 3-phosphate dehydrogenase (GAPDH).

### Chromatin immunoprecipitation

In brief, 1% formaldehyde solution was added to the cell culture medium for 10 min at 37 °C. Cells were washed three times with cold PBS and then resuspended in lysis buffer (1% SDS, 10 mM EDTA, and 50 mM Tris-HCl, pH 8.1) supplemented with 1 mM phenylmethylsulfonyl fluoride (PMSF). After a brief sonication, the lysates were cleared by centrifugation and resuspended in dilution buffer (0.01% SDS, 1% Triton X-100, 1.2 mM EDTA, 16.7 mM Tris-HCl, pH 8.1, and 167 mM NaCl) containing PMSF. Then, the lysates were incubated with an anti-p53 antibody overnight at 4 °C, and immune complexes were precipitated with Protein A/G Plus Agarose. The precipitates were sequentially washed with low-salt wash buffer (0.1% SDS, 1% Triton X-100, 2 mM EDTA, 20 mM Tris-HCl, pH 8.1, and 150 mM NaCl), high-salt wash buffer (0.1% SDS, 1% Triton X-100, 2 mM EDTA, 20 mM Tris-HCl, pH 8.1, and 500 mM NaCl), and LiCl wash buffer (0.25 M LiCl, 1% NP-40, 1% deoxycholate, 1 mM EDTA, and 10 mM Tris-HCl, pH 8.1). After the final wash, elution buffer (1% SDS and 0.1 M NaHCO3) was added, followed by incubation at room temperature (RT) for 15 min with rotation. Then, formaldehyde crosslinking was reversed by adding 0.3 M NaCl and heating at 65 °C for 4 h. Next, proteinase K was added, followed by incubation at 45 °C for 1 h. Then, DNA was recovered by phenol–chloroform extraction and ethanol precipitation. The resulting DNA pellets were resuspended in TE buffer and subjected to PCR using primers targeting the Ell3 promoter. The PCR products were separated by agarose gel electrophoresis.

### Luciferase assay

The 5′ upstream regulatory region of the Ell3 gene (− 1260 ~ + 493), which has one potential p53 binding site (RRRC(A/T)(A/T)GYYYnnnRRRC(A/T)(A/T)GYYY) (− 1113 ~ − 1093), was PCR-amplified with the following primers: forward primer: 5′-CTC GAG CGC GCC TGG CCT TTT T-3′ and reverse primer: 5′-AGA TCT GTT GAG CCT GAG CAG TAA GA-3′. The resulting fragment was then cloned into the pGL3 luciferase reporter vector (Promega, Madison, WI, USA) to construct the reporter plasmid for the luciferase assay. Next, 5 × 10^5^ 293 T cells were seeded in each well of a six-well tissue plate and cotransfected with the pGL3 reporter plasmid, pGL3-Basic plasmid, and pGL3-control plasmid (Promega) to normalize the transfection efficiency per well according to the manufacturer’s instructions. Firefly luciferase activity was measured in cell lysates 24 h after transfection using the Luciferase Assay System (cat no. E6110, Promega). Experiments were repeated at least three times with three replicates per sample. Reporter plasmids of the truncated forms of the Ell3 promoter were constructed by cloning the PCR product amplified with the following primers: Ell3Del #1 − 793 ~ + 493, forward primer: 5′-CTC GAG CAG TTA CTC GGG AGG CT-3′ and reverse primer: 5′-AGA TCT GTT GAG CCT GAG CAG TAA GA-3′; Ell3Del #2 − 214 ~ + 493, forward primer: 5′-CTC GAG GTT TAG GCC ACG AGG TGA-3′ and reverse primer: 5′-AGA TCT GTT GAG CCT GAG CAG TAA GA-3′.

### LIVE/DEAD assay

The LIVE/DEAD assay (Calcein AM & Ethidium Homodimer-1: cat no. L3224, Thermo Fisher Scientific) was used to evaluate the induction of apoptosis following Ell3 transfection. Twenty-four hours after transfection with a control or Ell3-expressing plasmid, the cells were incubated with Calcein AM (2 μM) and Ethidium Homodimer-1 (4 μM) for 30 min and observed under a fluorescence microscope.

### Annexin V/PI staining and cell cycle analyses

Cells washed twice with cold PBS were mixed with Annexin V-FITC and propidium iodide (PI) solution and incubated in the dark at RT for 15 min. The samples were analyzed by a Thermo Fisher flow cytometer. To analyze the cell cycle, cells were fixed with cold 70% ethanol for 2 h and then stained with PI (cat no. p3566, Thermo Fisher Scientific) at a final concentration of 50 μg/ml in the presence of 20 μg/ml RNase and 10% Triton X-100 in PBS for 1 h at 37 °C. Cells were washed with PBS, and the cell cycle was then evaluated by flow cytometry.

### Immunofluorescence staining

Cells were cultured on a slide and then fixed with 4% formaldehyde (cat no. F8775 Sigma-Aldrich) diluted in DPBS (cat no. 14190144 Thermo Fisher Scientific). Cells were washed three times with PBS, permeabilized with 0.1% Tween-20 for 30 min, and blocked for 30 min with blocking buffer (5% bovine serum albumin). After an overnight incubation with the primary antibodies at 4 °C, the culture slides were washed three times with PBS and incubated with fluorescent secondary antibodies for 1 h in the dark at RT. The culture slides were then washed three times in PBS and mounted with a DAPI-containing mounting solution (H-1200, Vector Laboratories, CA, USA). Images were examined with a fluorescence microscope (Eclipse TE2000, Nikon Metrology, Brighton, MI, USA).

### Senescence-associated β-galactosidase activity

The Cellular Senescence Assay Kit (Cat no CBA-230, Cell Biolabs, CA, USA) was used to stain β-galactosidase-positive cells. Cells were washed twice with PBS and fixed with fixing solution at RT for 5 min. Then, the cells were washed with PBS three times and further incubated in working solution for 4 h at 37 °C. Cells were washed with PBS, and blue staining was then observed under a light microscope.

### Statistical analysis

Each experiment was performed at least three times. Statistical significance between two groups was determined using Student’s *t* test, and a *P* value of < 0.05 was considered significant. All statistical analyses were performed using the SAS statistical package v.9.13 (SAS Institute, Cary, North Carolina, USA).

## Results

### Suppression of Ell3 expression induces stem cell senescence

**Fig. 1:**
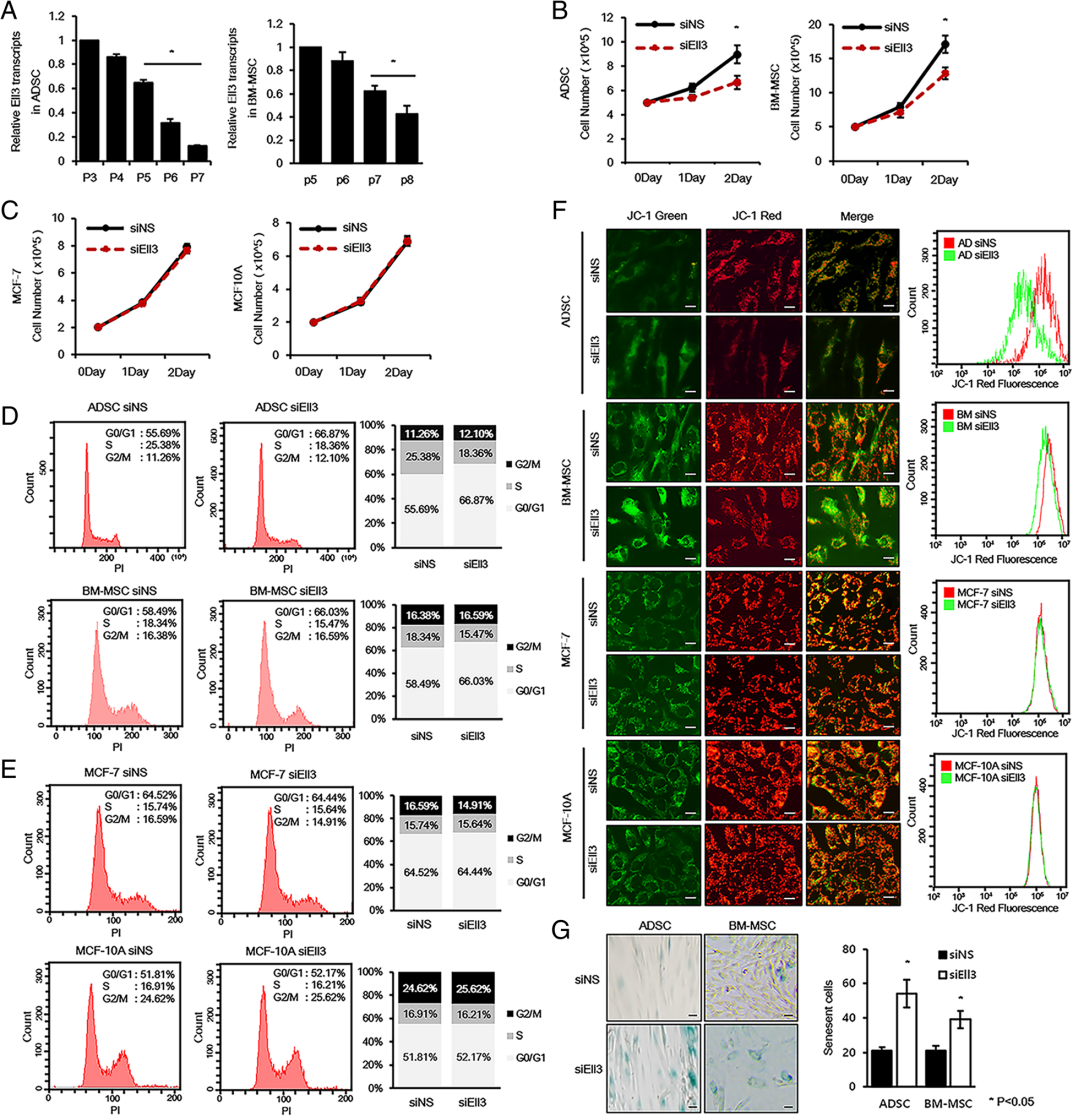
Suppression of Ell3 expression induces stem cell senescence. **a** Quantitative reverse transcription PCR (qRT-PCR) analysis was performed on ADSCs and BM-MSCs at the indicated culture passage. The numbers of **b** ADSCs and ES-MSCs as well as those of **c** MCF7 and MCF10A cells transfected with siNS or siEll3 were counted on the indicated days after transfection. Cell cycle analysis of **d** ADSCs and BM-MSCs as well as **e** MCF7 and MCF10A cells transfected with siNS or siEll3 was performed by FACS 48 h after siRNA transfection (left panel). Quantitation of the cell cycle analysis results is presented as a graph (right panel). **f** The mitochondrial membrane potentials of ADSCs, BM-MSCs, MCF7 cells, and MCF10A cells transfected with siNS or siEll3 were evaluated by JC-1 staining (left) and flow cytometry analysis (right). Scale bar 25 μm. **g** β-gal staining was performed with ADSCs and BM-MSCs transfected with siNS or siEll3. β-gal (+) cells were imaged under a light microscope (left) and quantified (right). Scale bar 20 μm. The experiments were repeated three times independently, and the results presented as bars represent the mean ± s.d. Abbreviations: siNS, nonspecific siRNA; siEll3, siRNA targeting Ell3; ADSCs, adipose stem cells; BM-MSCs, bone marrow-derived mesenchymal stem cells

To study the roles of Ell3 on the senescence of adult stem cells, we first examined the passage-dependent expression pattern of Ell3 in ADSCs and bone marrow-derived stem cells (BM-MSCs). As shown in Fig. 1a, Ell3 expression decreased as the in vitro culture passage of ADSCs and BM-MSCs increased. Because cell proliferation is reduced with culture passaging, we examined whether the Ell3 expression level is associated with the proliferation rate of stem cells. When Ell3 expression was suppressed by the transfection of siEll3 into ADSCs and BM-MSCs, cell proliferation was significantly slowed in both types of stem cells (Fig. 1b). On the other hand, the transfection of siEll3 into other cell types, such as MCF7 and MCF10a cells, had no effect on cell proliferation, indicating that the effect of Ell3 expression on proliferation is indigenous to stem cells (Fig. 1c). The distinct function of Ell3 in stem cell proliferation was further supported by cell cycle analysis. Ell3 suppression resulted in an increased number of ADSCs and BM-MSCs in the G0/G1 phase (Fig. 1d). Cell cycle alteration was not detected in Ell3-suppressed MCF7 or MCF10a cells (Fig. 1e).

We next examined the effect of Ell3 knockdown on the mitochondrial activity by analyzing mitochondrial membrane potential by JC-1 staining. Figure 1f and Additional file 2: Figure S1 indicate that the mitochondrial membrane potential was significantly decreased by the suppression of Ell3 in ADSCs and BM-MSCs, whereas the mitochondrial activity in MCF7 and MCF10a cells was not disturbed by the suppression of Ell3. Next, we questioned whether the changes in the cell proliferation rate, cell cycle pattern, and mitochondrial membrane potential following siEll3 transfection were caused by stem cell senescence. As expected, siEll3-transfected ADSCs and BM-MSCs exhibited higher levels of senescent cells than the control, as measured by β-gal staining (Fig. 1g). Consistent with the results of cell proliferation, cell cycle, and mitochondrial membrane potential analyses, the number of β-gal (+) cells was not significantly increased in siEll3-transfected MCF7 or MCF10a cells (data not shown). Taken together, our results show that the Ell3 expression level is associated with stem cell senescence, and knockdown of Ell3 facilitates the cellular senescence of adult stem cells.

### Ell3 regulates the balance between osteogenic and adipogenic differentiation of stem cells

**Fig. 2:**
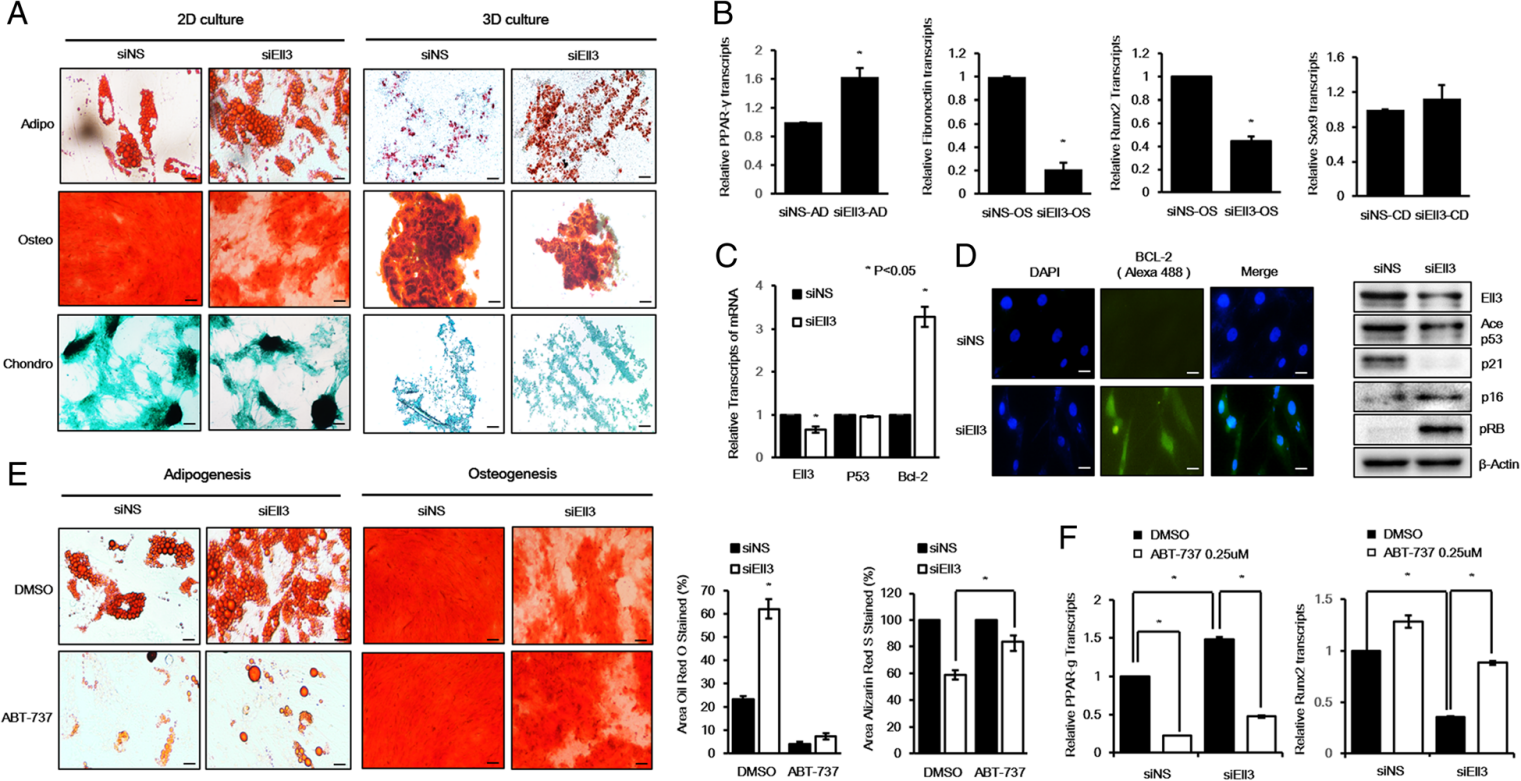
Ell3 suppression shifts the differentiation potential of ADSCs into adipogenesis by inducing Bcl-2 accumulation. **a** The adipogenic, osteogenic, and chondrogenic lineage differentiation efficiency of ADSCs transfected with siNS or siEll3 was evaluated by oil red O staining, Alcian Blue staining, and alizarin red S staining, respectively, under 2D culture and 3D pellet cultures. The cells were then cultured for 2 weeks (adipogenic) and 3 weeks (osteogenic, chondrogenic), and the medium was changed every 2 days. Scale bar 20 μm. **b** The expression of each lineage marker gene in ADSCs transfected with siNS or siEll3 was analyzed by qRT-PCR and cultured in 2D differentiation condition for 48 h. **c** The expression of Ell3, p53, and Bcl-2 in ADSCs transfected with siNS or siEll3 for 48 h was analyzed by qRT-PCR (left) and immunocytochemical staining (right). **d** The protein levels of Ell3, p53, p21, p16, and phospho-Rb in ADSCs transfected with siNS or siEll3 for 48 h were analyzed by immunoblot (right). β-actin was used as the loading control. Scale bar 25 μm. **e** The effect of ABT-737 treatment on the adipogenic and osteogenic lineage differentiation efficiencies of ADSCs transfected with siNS or siEll3 was evaluated by oil red O staining and alizarin red S staining under 2D culture (left). Adipogenesis was quantified by the extracting oil red O stain from cells according to the protocol, and osteogenesis was quantified using the ImageJ program (right). The cells were cultured for 2 weeks (adipogenic differentiation) and 3 weeks (osteogenic differentiation), and the medium containing 0.25 μM ABT-737 was changed every 2 days. Scale bar 25 μm. **f** ADSCs transfected with siNS or siEll3 for 48 h were treated with 0.25 μM ABT-747 for 24 h, and the expression of PPAR-γ and Runx2 was detected by qRT-PCR. The experiments were repeated three times independently, and the results presented as bars represent the mean ± s.d. Abbreviations: siNS, nonspecific siRNA; siEll3, siRNA targeting Ell3; ADSCs, adipose stem cells; ES-MSCs, embryonic stem cell-derived mesenchymal-like stem cells

As a common progenitor of osteoblasts and adipocytes, MSCs are delicately well balanced for their commitment toward osteogenic and adipogenic differentiation. As senescence is a biological process that has been linked to disturbances in lineage differentiation efficiency [20–22], we hypothesized that Ell3-suppressed ADSCs show a differentiation pattern of aged stem cells. As expected, the suppression of Ell3 resulted in dysregulation of the adipo-osteogenic balance in ADSCs, and Ell3-suppressed ADSCs showed the typical differentiation pattern of senescent stem cells, enhanced adipogenesis, and decreased osteogenesis in both 2D and 3D differentiation conditions (Fig. 2a). The expression of PPAR-γ, adiponectin, 4-1BB, fibronectin, and Runx2, representative marker genes of adipogenesis and osteogenesis, further supports that Ell3 suppression shifts the adipo-osteogenic differentiation of ADSCs (Fig. 2b, Additional file 3: Figure S2). Bcl-2 is an antiapoptotic protein that accumulates in senescent cells at high levels [23, 24]. Therefore, we next examined whether Ell3 suppression affects Bcl-2 expression. As shown in Fig. 2c, Bcl-2 expression was significantly increased at both the RNA and protein level when Ell3 expression was suppressed. Consistent with the Bcl-2 accumulation, the p53-p21 pathway, which functions to inhibit Bcl-2 activity [25, 26], was suppressed in siEll3 ADSCs (Fig. 2d). Furthermore, phospho-retinoblastoma and p16, which play a key role in senescence, were significantly accumulated in the Ell3-depleted ADSCs. We next questioned whether the clearance of Ell3-suppressed senescent ADSCs with ABT-737, a senolytic drug that can selectively kill Bcl-2-positive cells [27], could restore the adipo-osteogenic balance. Figure 2e shows that Ell3 suppression enhanced adipogenesis of ADSCs up to 3-fold compared to controls (siNS vs. siEll3 in the presence of DMSO). However, treatment with ABT-737 resulted in 2-fold increases of Ell3-suppressed ADSCs compared to controls (siNS vs. siEll3 in the presence of ABT-737). In contrast to adipogenesis, ABT-737 treatment increased the osteogenic differentiation efficiency of siEll3-transfected ADSCs (Fig. 2e, Additional file 4: Figure S3). Consistent with the phenotype, ABT-737 treatment significantly decreased PPAR-gamma expression and increased Runx2 expression (Fig. 2f). Together, our results suggested that Ell3 expression is linked to the senescence and adipo-osteogenic differentiation balance of ADSCs.

### Expression of supra-physiological Ell3 levels induces stem cell apoptosis

**Fig. 3:**
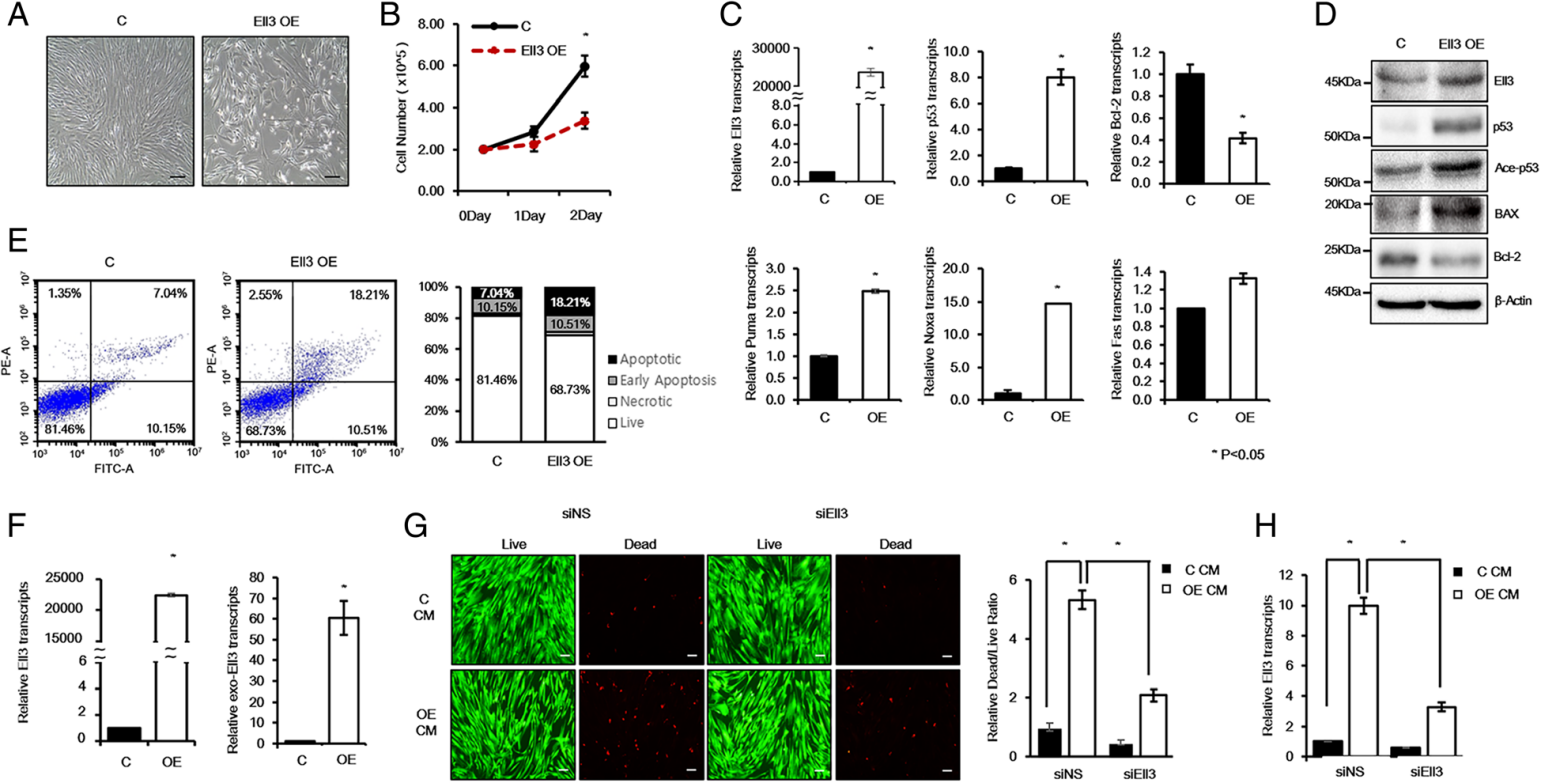
Ectopic Ell3 expression induces ADSC apoptosis. **a** A control or Ell3-expressing plasmid was transfected into ADSCs by lipofectamine, and cell morphology was observed under a light microscope 48 h after transfection. Scale bar 50 μm. **b** The numbers of ADSCs transfected with the control or Ell3-expressing plasmid were counted on the indicated days after transfection. **c** The effect of ectopic Ell3 expression on the expression of the indicated genes was evaluated by qRT-PCR analysis. The expression of each gene in ADSCs transfected with the control plasmid was used as the control. **d** Immunoblot analysis was performed to analyze the effect of ectopic Ell3 expression on the protein accumulation of each gene. **e** The apoptosis of ADSCs transfected with the control or Ell3-expressing plasmid was analyzed by Annexin V staining and flow cytometric analysis. **f** Relative expression of total Ell3 and exogenic Ell3 was evaluated by qRT-PCR. The expression of exogenic Ell3 was analyzed by qRT-PCR with primers targeting the 5′ UTR of the Ell3 expression plasmid (forward primer: 5′-ATC CAC TAG TCC AGT GTG GT-3′, reverse primer: 5′-TGA AGG AGA GGC AGG AC-3′). **g** Live and dead staining was performed with ADSCs cultured in conditioned media prepared from culture media from ADSCs transfected with the control or Ell3-expressing plasmid. Recipient cells were either siNS- or siEll3-transfected ADSCs. Live (green) and dead [6] cells were imaged under a light microscope (left). The relative ratio of live and dead cells was evaluated by counting stained cells and presented in a graph (right). Scale bar 20 μm. **h** The relative expression level of Ell3 in the ADSCs from the experiments in **g** was analyzed by qRT-PCR. The experiments were repeated three times independently, and the results presented as bars represent the mean ± s.d. Abbreviations: C, control plasmid; Ell3 OE, Ell3-expressing plasmid; C-CM, conditioned media prepared from the culture media of ADSCs transfected with the control plasmid; OE-CM, conditioned media prepared from the culture media of ADSCs transfected with the Ell3-expressing plasmid; siNS, nonspecific siRNA; siEll3, siRNA targeting Ell3; ADSCs, adipose stem cells; BM-MSCs, bone marrow-derived mesenchymal stem cells

Because Ell3-suppressed stem cells showed the senescence phenotype and their lineage differentiation was primed to adipogenic differentiation, we next questioned whether the ectopic expression of Ell3 has the reverse effect on stem cells in terms of senescence and lineage differentiation. Contrary to our expectation, ADSCs showed severe cell damage in terms of cell morphology upon transient transfection of the Ell3-expressing plasmid (Fig. 3a). Unlike the ADSCs transfected with the control plasmid, which showed normal proliferation, the number of ADSCs transfected with the Ell3-expressing plasmid gradually decreased following the transfection (Fig. 3b). To determine whether the decrease in the number of Ell3-overexpressing ADSCs was due to apoptosis, the expression of representative apoptotic genes was analyzed. As expected, apoptosis-related genes, such as p53, NOXA, and PUMA, were highly expressed, whereas the expression of BCL-2, a senescence marker gene, was significantly decreased in Ell3-overexpressing ADSCs (Fig. 3c). Immunoblot analysis further supported the accumulation of apoptosis-related proteins in Ell3-overexpressing ADSCs (Fig. 3d). Transfection of the Ell3-expressing plasmid into BM-MSCs increased the number of apoptotic cells, whereas MCF7 cells did not show any notable phenotypic changes upon Ell3 overexpression (Additional file 5: Figure S4), indicating that the effect of Ell3 overexpression on the initiation of apoptosis might be stem cell-specific. We next measured the apoptotic population in ADSCs transfected with the control or Ell3-expressing plasmid by Annexin V-FITC and PI staining and FACS analysis. Consistent with the marker expression pattern, the Annexin V (+)/PI (+) cell population was significantly increased in Ell3-overexpressing ADSCs (Fig. 3e). Unlike stem cells, other cell types, such as 293 T, MCF7, and MCF10a cells, did not show the apoptotic phenotype upon Ell3 overexpression (data not shown). Because ectopic Ell3 overexpression induced an apoptotic response in stem cells, we quantitatively analyzed the total Ell3 expression level in ADSCs. Intriguingly, the total Ell3 level was increased up to ~ 20,000-fold (Fig. 3f, left panel). Considering the lipofectamine-mediated transfection efficiency of ADSCs, which is known to be less than 10%, the expression level of Ell3 was unexpectedly high. Therefore, we analyzed the expression of ectopic Ell3 using specific Ell3 primer sets targeting exogenous Ell3 transcribed from the transfected Ell3 plasmid. As expected, the ectopic Ell3 level expressed by the transfected plasmid was increased only ~ 60-fold (Fig. 3f, right panel). The high level of total Ell3 transcripts compared to the exogenous Ell3 expression level suggests that most Ell3 is produced not by ADSCs transfected with the Ell3 plasmid but rather by neighboring transfected cells. To verify this possibility, we cultured ADSCs with conditioned media from control or Ell3 plasmid-transfected ADSCs and performed live and dead cell staining. As expected, the dead cell population was increased in ADSCs cultured in the conditioned media from Ell3-overexpressing ADSCs (Fig. 3g). Of note, Ell3-suppressed recipient ADSCs showed a significantly low population of dead cells compared to control ADSCs in the conditioned media from Ell3-overexpressing ADSCs (Fig. 3g). In addition, endogenous Ell3 expression was increased in the recipient ADSCs when stem cells were treated with conditioned media from Ell3-overexpressing ADSCs, and the increase in the Ell3 transcript level in Ell3-suppressed recipient ADSCs was significantly lower than that in control ADSCs (Fig. 3h). The results shown in Fig. 3g and h indicate that the endogenous Ell3 expression level is positively correlated with stem cell apoptosis. Together, our results showed that endogenous Ell3 expression above the physiological level results in the apoptosis of stem cells.

### p53 functions as a transcriptional activator of Ell3 during stem cell apoptosis

**Fig. 4:**
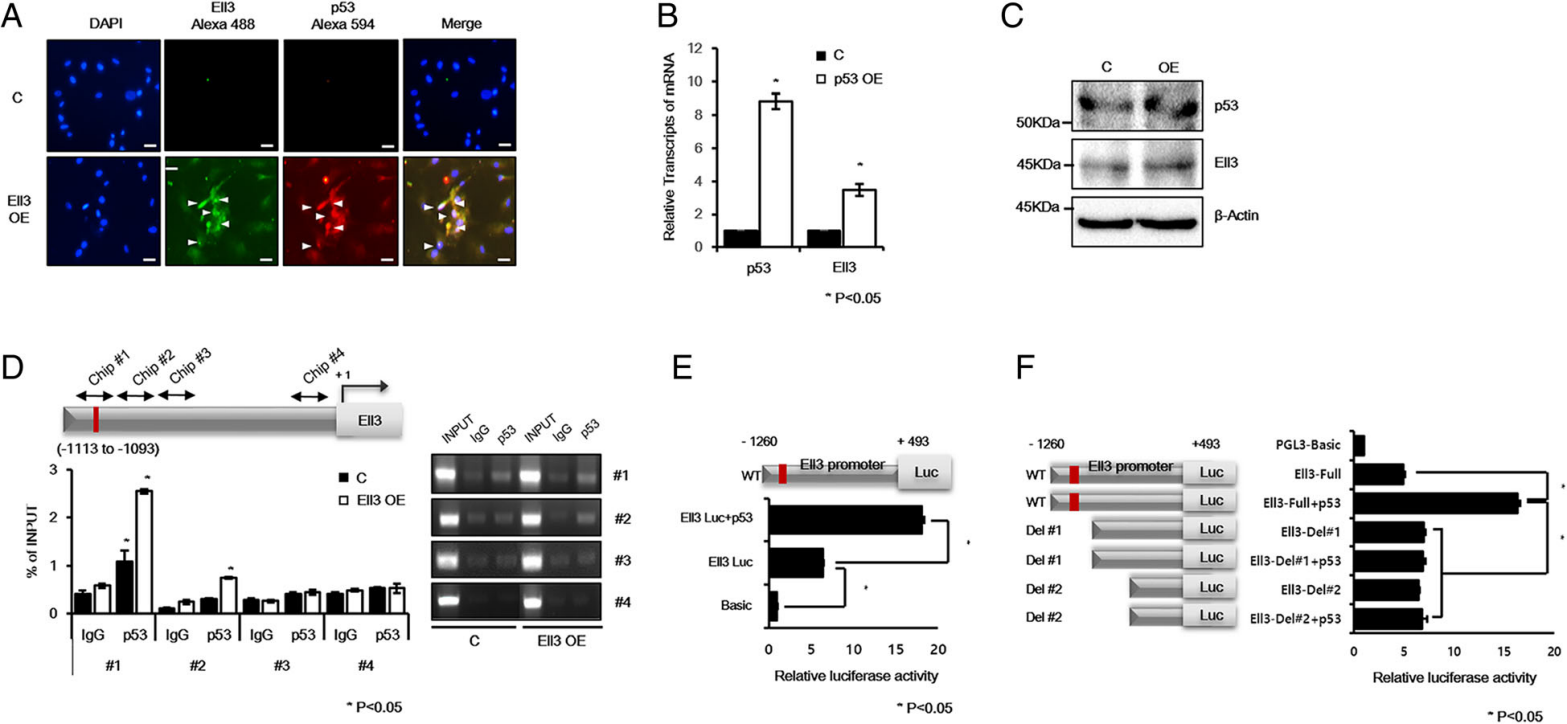
p53 directly activates Ell3 transcription. **a** The accumulation of Ell3 and p53 protein was examined by the immunocytochemical staining of ADSCs transfected with the control or Ell3-expressing plasmid. Scale bar 50 μm. The relative expression of p53 and Ell3 in ADSCs transfected with the control or Ell3-expressing plasmid was analyzed by **b** qRT-PCR or **c** immunoblot. **d** Chromatin immunoprecipitation was performed with control and Ell3 OE cells using antibodies against IgG and p53. The potential p53 binding site (RRRC(A/T)(A/T) GYYY nnnRRRC (A/T) (A/T) GYYY) (− 1113 ~ − 1093) is indicated in red on the ELL3 promoter. **e** Luciferase assay with the Ell3 promoter. Two hundred ninety-three T cells were transfected with the indicated plasmid for 24 h, and cell lysates were analyzed for luciferase activity. The pGL3-basic plasmid was used as the negative control. **f** Luciferase assay with deletion mutants of the Ell3 promoter. Deletion mutant-1 contains the promoter region of Ell3 from − 793 to + 493, and deletion mutant-2 contains the promoter region from − 214 to + 493. The experiments were repeated three times independently, and the results presented as bars represent the mean ± s.d. Abbreviations: C, control plasmid; Ell3 OE, Ell3-expressing plasmid; p53 OE, p53-expressing plasmid; WT, Ell3 promoter from − 1260 to + 493; Del #1, Ell3 promoter from − 793 to + 493; Del #2, Ell3 promoter from − 214 to + 493; Luc; luciferase gene

After observing that the activation of endogenous Ell3 expression is correlated with the apoptosis of stem cells, we wanted to further study the mechanism underlying Ell3 expression. We noted that significant amounts of p53 and Ell3 accumulated in the nuclei of Ell3-transfected ADSCs (Fig. 4a). As p53 is a representative inducer of apoptosis, we questioned whether p53 expression is correlated with endogenous Ell3 expression in ADSCs. To test this, we examined the effect of p53 overexpression on Ell3 expression in ADSCs. The results illustrated in Fig. 4b and c show that transient transfection of the p53-expressing plasmid increased Ell3 expression at both the RNA and protein level. Because potential p53 binding sequences exist at − 1113 bp and − 1093 bp on the Ell3 promoter, we tested whether p53 directly binds to this sequence. Using chromatin immunoblot analysis and qRT-PCR in combination, we confirmed that p53 directly binds to the expected sequence, and the binding was increased when the Ell3 level was increased by exogenous Ell3 overexpression (Fig. 4d). Next, we performed luciferase analysis to confirm that p53 activates the Ell3 promoter. As shown in Fig. 4e, the Ell3 promoter activity was significantly increased in the presence of p53, which indicates that p53 functions as a transcriptional activator of the Ell3 promoter. To further confirm that p53 activates the Ell3 promoter by binding to the potential binding site, we constructed deletion mutants of the Ell3 promoter and tested their activities by luciferase assay. As expected, the promoter activity of deletion mutant-1, in which the p53 binding site was deleted, was significantly decreased compared to that of the full-length promoter (Fig. 4f). Taken together, our results demonstrate that p53 directly binds to the Ell3 promoter to enhance transcriptional expression of Ell3.

## Discussion

RNA polymerase II elongation factor Ell3 is reported to perform distinct functions in embryonic stem cells and cancer cells [16, 28, 29]. For example, Ell3 is involved in the differentiation efficiency of embryonic stem cells, whereas Ell3 regulates anticancer drug responses in breast cancer cells. These reports suggest that the biological function of Ell3 strongly depends on the intercellular context of each cell type.

**Fig. 5:**
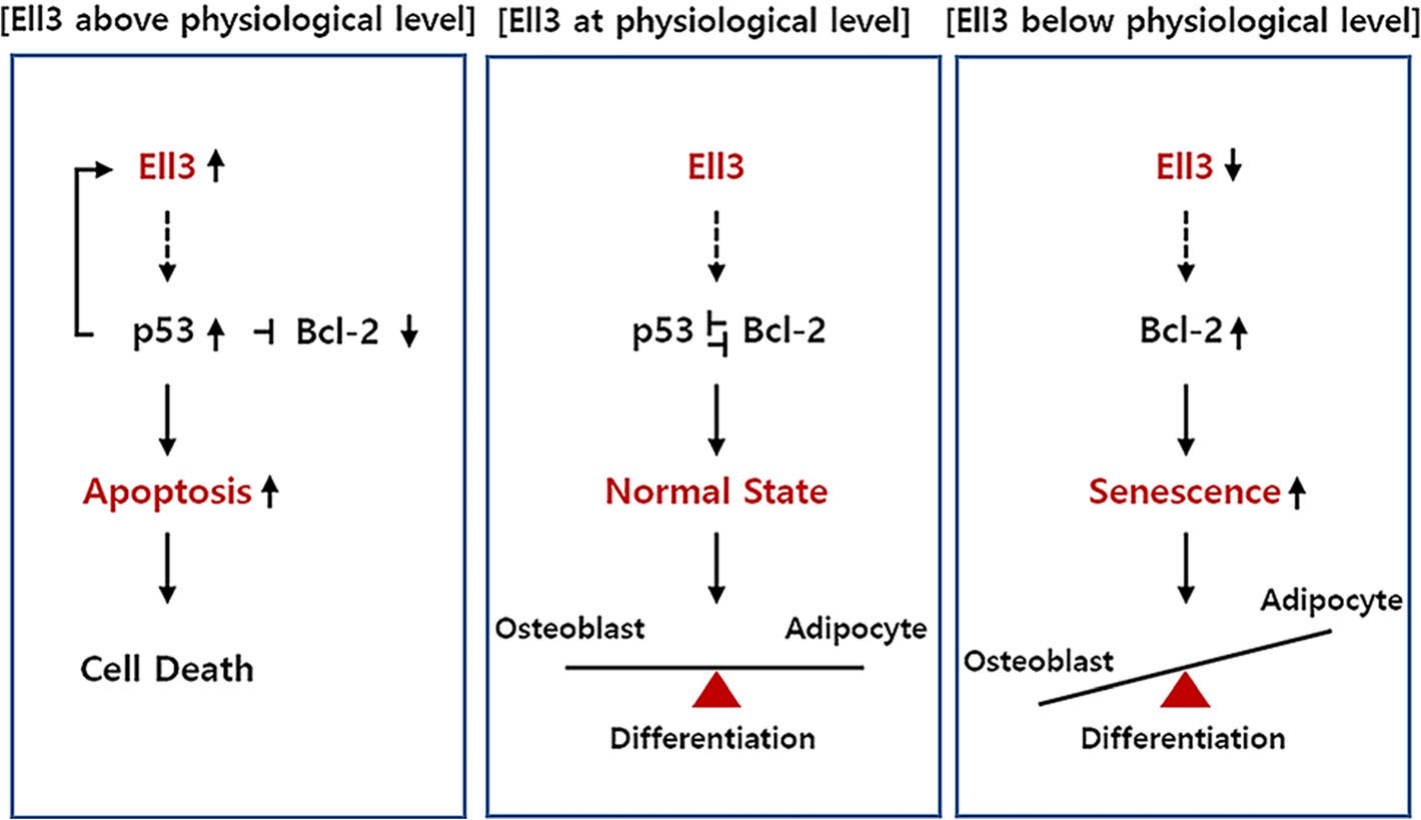
Proposed model of Ell3 activity in somatic stem cells

In this study, we present a model of novel Ell3 function in somatic stem cells to regulate senescence and apoptosis (Fig. 5). We show that suppression of Ell3 expression triggers ADSC senescence, demonstrated by cell cycle alteration, decreased mitochondrial activity, and increased numbers of β-gal (+) cells. Annexin V staining and FACS analysis revealed that Ell3 suppression did not result in apoptosis of ADSCs (Additional file 6: Figure S5). We demonstrated that Ell3 suppression disturbs the lineage differentiation efficiency to inhibit osteogenic differentiation and enhance adipogenic differentiation. Furthermore, we also confirmed that treatment with a senolytic drug targeting the senescence of ADSCs that highly express Bcl-2 restores the imbalanced differentiation efficiency of Ell3-suppressed ADSCs compared to that in control cells. Contrary to the effect of Ell3 suppression on inducing ADSC senescence, the ectopic expression of Ell3 triggers cell apoptosis. Using the chromatin precipitation and luciferase assays, we demonstrated that p53 functions as a direct transcriptional activator of Ell3, revealing that the function of Ell3 is related to the p53-Bcl2 axis in both senescent and apoptotic ADSCs.

Several reports suggest that Ell3 activity is associated with p53 protein stability. In mouse embryonic stem cells, Ell3 promotes p53 degradation, which leads to the protection of differentiating cells and enhancement of differentiation efficiency [28]. On the other hand, Ell3 stabilizes the p53 protein in anticancer drug-treated breast cancer cells by regulating the proteasomal degradation pathway [29]. The suppression of Ell3 decreases p53 protein expression without changing its transcription level in ADSCs, indicating that similar to breast cancer cells, Ell3 regulates p53 protein stability in ADSCs.

An interesting novel finding of our work is that p53 functions as a direct transcriptional activator of Ell3, which partially explains the mechanism by which Ell3 is involved in the apoptosis of ADSCs. While p53 is also transcriptionally activated by ectopic Ell3 expression (Fig. 3c), whether Ell3 directly activates p53 transcription remains inconclusive. We noted that total Ell3 expression was increased up to ~ 20,000-fold in ADSCs undergoing apoptosis by ectopic Ell3 expression, while p53 transcripts were increased only ~ 8-fold. Chromatin immunoprecipitation analysis did not reveal any association between Ell3 and the p53 promoter (data not shown). Therefore, it is plausible to presume that the effect of Ell3 on p53 transcription is indirect.

Another notable finding is that only ~ 3% of accumulated Ell3 transcripts were exogenic Ell3 transcribed from the transfected Ell3 plasmid, which is a reasonable result considering the low transfection efficiency of ADSCs and suggests that most Ell3 transcripts originate from the endogenous Ell3 gene in ADSCs undergoing apoptosis. Based on these results, we speculate that small populations of ADSCs that are transfected by an Ell3-expressing plasmid and express Ell3 above the physiological level trigger neighboring cells to initiate the apoptotic process by secreting apoptosis-inducing molecules. Indeed, ADSCs cultured in the conditioned media of Ell3-transfected ADSCs also showed the apoptotic phenotype, indicating that conditioned media contains unknown molecules to stimulate the apoptosis of recipient ADSCs. More importantly, depletion of Ell3 in the recipient ADSCs inhibited the apoptotic effect of conditioned media. Taken together, we suggest that Ell3 functions as a switch to turn on the apoptotic process in ADSCs.

p53 physically interacts and inhibits antiapoptotic Bcl-2 activity during the p53-dependent apoptotic process [30]. The fact that p53 protein expression was decreased and Bcl-2 transcription was increased in Ell3-suppressed ADSCs (Fig. 2c and d) implies that p53 might function as a transcriptional repressor of Bcl-2 expression. While one study reported that p53 binds to the Bcl-2 promoter [31], this has not been confirmed, and whether p53 directly represses Bcl-2 expression remains unknown. Contrary to substantial evidence supporting the role of p53 as an ultimate direct transcriptional activator via its two functionally specialized transactivation domains, several lines of evidence support that the transcriptional repressive activity of p53 might be indirect [32, 33]. Therefore, how depletion of the p53 protein in Ell3-suppressed ADSCs is associated with the transcriptional activation of Bcl-2 remains unclarified. We expect that further studies on the molecular mechanism underlying the linkage among Ell3, p53, and Bcl-2 will address how Ell3 regulates stem cell-specific senescence and apoptosis.

## Conclusions

Ell3 functions as a critical decision maker at the crossroad between senescence and apoptosis by regulating p53-Bcl2 axis in the somatic stem cells.

## Additional files

Additional file 1: **Table S1.** The primers used in performing qRT-PCR. (DOCX 21 kb)

Additional file 2: **Figure S1.** JC-1 staining results of the mitochondrial membrane potentials of ADSCs, BM-MSCs, MCF7 cells, and MCF10A cells transfected with siNS or siEll3 were quantified by the ImageJ program. (PDF 613 kb)

Additional file 3: **Figure S2.** The expression of indicated markers of adipogenic (AD) and chondrogenic (CD) differentiation was analyzed by quantitative RT-PCR. The experiments were repeated three times independently, and the results presented as bars represent the mean ± s.d. (PDF 365 kb)

Additional file 4: **Figure S3.** The effect of ABT-737 treatment on the osteogenic lineage differentiation efficiencies of ADSCs transfected with siNS or siEll3 was evaluated by Alizarin Red S staining under 2D culture. The cells were cultured for 3 weeks, and the medium containing 0.25 μM ABT-737 was changed every 2 days. (PDF 1549 kb)

Additional file 5: **Figure S4.** The effect of Ell3 overexpression on MCF7 cells and BM-MSCs. Live and dead staining was performed on MCF7 cells and BM-MSCs transfected with the control or Ell3-expressing plasmid. Live (green) and dead [6] cells were imaged 48 h after transfection under a light microscope (left). The relative ratio of live and dead cells was evaluated by counting stained cells and presented as a graph (right). The experiments were repeated three times independently, and the results presented as bars represent the mean ± s.d. (PDF 1495 kb)

Additional file 6: **Figure S5.** Apoptosis of ADSCs transfected with siNS or siEll3 was analyzed by Annexin V staining and flow cytometry. (PDF 1103 kb)

## Abbreviations

ADSCs: Adipose-derived stem cells
BM-MSCs: Bone marrow-derived stem cells
EGF: Epidermal growth factor
Ell: Elven-nineteen lysine-rich leukemia
MEF: Mouse embryonic fibroblasts
MSCs: Mesenchymal stem cells
PI: Propidium iodide
SASP: Senescence-associated secretory phenotype
SDS–PAGE: Sodium dodecyl sulfate-polyacrylamide gel electrophoresis
