# Supplementary material for: Ell3 functions as a critical decision maker at the crossroad between stem cell senescence and apoptosis
Source: Stem Cell Res Ther. 2019 Jan 17;10:32. doi: 10.1186/s13287-019-1137-9 (PMC6335702; doi:10.1186/s13287-019-1137-9)
Supplement: Supplementary file 1 — Table S1. The primers used in performing qRT-PCR. (DOCX 21 kb) [file 13287_2019_1137_MOESM1_ESM.docx]

**Table S1**

| Primer | Human Sequence (5’ to 3’) | |
| --- | --- | --- |
| GAPDH | F | ACC CAG AAG ACT GTG GAT GG |
|  | R | TCT AGA CGG CAG GTC AGG TC |
| Ell3 | F | CTA CAA GGC CTG ACC AAT CAG G |
|  | R | CTG GAG TTC CTC GCC GAA c |
| p53 | F | AGCTAGCTCAGGATGACATTGATG |
|  | R | GCCGATGGGCTGGACAG |
| Bcl-2 | F | TCT CCT GAC ATT GAC CTT GGC |
|  | R | CAA GGT GCT GGC TGA GTA GAT C |
| p21 | F | ATT CCA TGG AGC CAG GCT TTC |
|  | R | CAT TTG GGT CAA ACT CCA ACT GTG |
| p16 | F | TTG ACA GCG ACA AGA AGT GG |
|  | R | GCC ATT CAC GTC GTC CTT AT |
| NOXA | F | ATTTTGGCCCTCTCTTCCA |
|  | R | TTGTCCGCTGCAAAGAAGTA |
| PUMA | F | TCC CAG GAA TTG GTG ATA AAG TAG A |
|  | R | CTG GCA TGA CGC GAA CAA TA |
| FAS | F | GAG ATG AGC TTC CTA CAG CAC |
|  | R | TCA CCG CCT CGG CTT GTC ACA T |
| Ell3 Luc WT | F | CTC GAG CGC GCC TGG CCT TTT T |
|  | R | AGA TCT GTT GAG CCT GAG CAG TAA GA |
| Ell3 Del #1 | F | CTC GAG CAG TTA CTC GGG AGG CT |
|  | R | AGA TCT GTT GAG CCT GAG CAG TAA GA |
| Ell3 Del #2 | F | CTC GAG GTT TAG GCC ACG AGG TGA |
|  | R | AGA TCT GTT GAG CCT GAG CAG TAA GA |
| Ell3 Chip #1 | F | CAG GGT CTC TCT CTG TTG CTC |
|  | R | TTT CTC TGC GGC AAT ATC CT |
| Ell3 Chip #2 | F | CAT CCC TGT AAT CCC AGC AC |
|  | R | AGT GCA GTG GCA CGA TCT C |
| Ell3 Chip #3 | F | GCA GGT CCA GGG ATT TAA CA |
|  | R | TCT CAC CTC GTG GCC TAA AC |
| Ell3 Chip #4 | F | TGG GGA TAG CTC TTC CGA TA |
|  | R | CCT CCT CTG TTC AGG GTT TG |
| Ell3 EXO | F | ATC CAC TAG TCC AGT GTG GT |
|  | R | TGA AGG AGA AGA GGC AGG AC |
| PPAR-γ | F | ATG ACA GCG ACT TGG CAA TAa |
|  | R | GCA ACT GGA AGA AGG GAA AT |
| Fibronectin | F | CAG TGG GAG ACC TCG AGA AG |
|  | R | TCC CTC GGA ACA TCA GAA AC |
| Sox9 | F | TAC GAC TAC ACC GAC CAC CA |
|  | R | TCA AGG TCG AGT GAG CTG TG |
| Sox6 | F | GAG GCA GTT CTT TAC TGT GG |
|  | R | CCG CCA TCT GTC TTC ATA C |
| Sox5 | F | CCC TTG CAT GTG AGT TTT CCC |
|  | R | TGC CTT CTG AGG TGA GGT AGA |
| Osteocalcin | F | CTG ACC TCA CAG ATG CCA A |
|  | R | GGT CTG ATA GTC TGT CAC AA |
| Adiponectin | F | TTC ACC GAT GTC TCC CTT AGG |
|  | R | GGC ATG ACC AGG AAA CCA C |
| Runx2 | F | ATT CCT GTA GAT CCG AGC ACC |
|  | R | GCT CAC GTC GCT CAT TTT GC |
| 4-1BB | F | GCT TTG GGA CAT TTA ACG AT |
|  | R | GCA GCT ACA GCC ATC TTC CT |
