# Supplementary figures and images for: Ell3 functions as a critical decision maker at the crossroad between stem cell senescence and apoptosis
Source: Stem Cell Res Ther. 2019 Jan 17;10:32. doi: 10.1186/s13287-019-1137-9 (PMC6335702; doi:10.1186/s13287-019-1137-9)

(A)

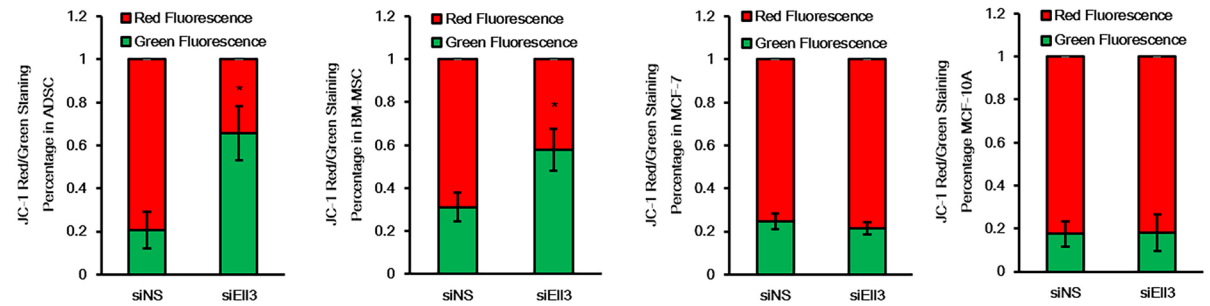

Supplementary Figure-1

Supplement: Supplementary file 2 — Figure S1. JC-1 staining results of the mitochondrial membrane potentials of ADSCs, BM-MSCs, MCF7 cells, and MCF10A cells transfected with siNS or siEll3 were quantified by the ImageJ program. (PDF 613 kb) [file 13287_2019_1137_MOESM2_ESM.pdf]

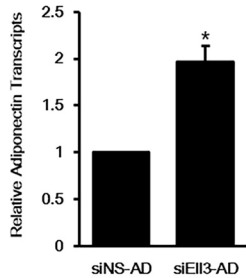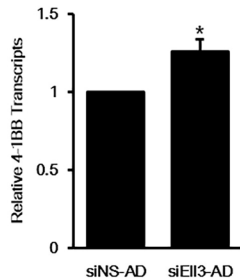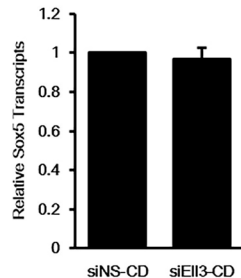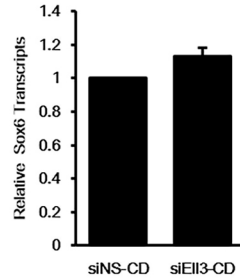

Supplementary Figure-2

Supplement: Supplementary file 3 — Figure S2. The expression of indicated markers of adipogenic (AD) and chondrogenic (CD) differentiation was analyzed by quantitative RT-PCR. The experiments were repeated three times independently, and the results presented as bars represent the mean ± s.d. (PDF 365 kb) [file 13287_2019_1137_MOESM3_ESM.pdf]

siNS

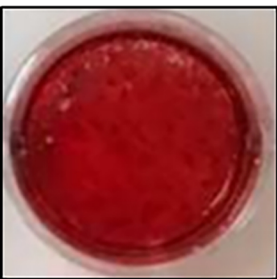

DMSO

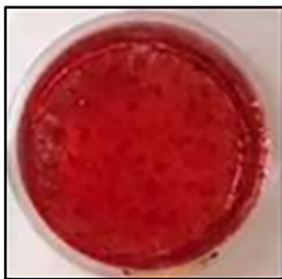

ABT-737  
0.25 $\mu$ M

siE113

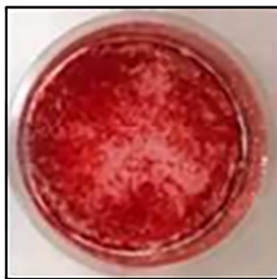

DMSO

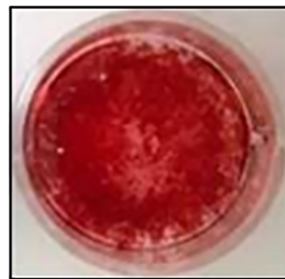

ABT-737  
0.25 $\mu$ M

Supplementary Figure-3

Supplement: Supplementary file 4 — Figure S3. The effect of ABT-737 treatment on the osteogenic lineage differentiation efficiencies of ADSCs transfected with siNS or siEll3 was evaluated by Alizarin Red S staining under 2D culture. The cells were cultured for 3 weeks, and the medium containing 0.25 μM ABT-737 was changed every 2 days. (PDF 1549 kb) [file 13287_2019_1137_MOESM4_ESM.pdf]

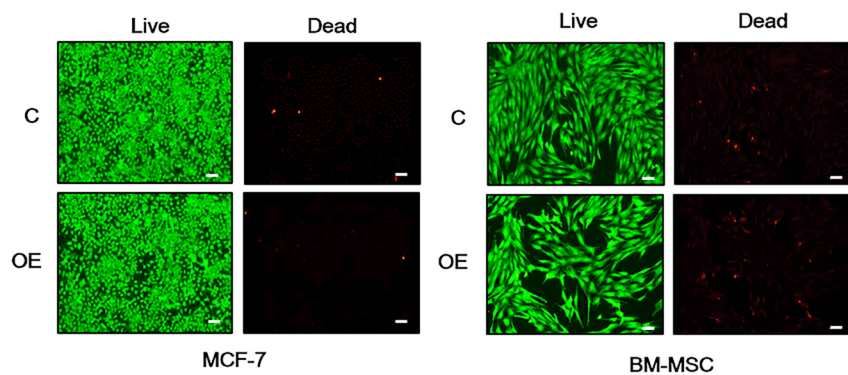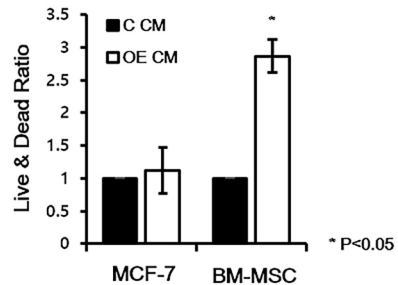

Supplementary Figure-4

Supplement: Supplementary file 5 — Figure S4. The effect of Ell3 overexpression on MCF7 cells and BM-MSCs. Live and dead staining was performed on MCF7 cells and BM-MSCs transfected with the control or Ell3-expressing plasmid. Live (green) and dead [6] cells were imaged 48 h after transfection under a light microscope (left). The relative ratio of live and dead cells was evaluated by counting stained cells and presented as a graph (right). The experiments were repeated three times independently, and the results presented as bars represent the mean ± s.d. (PDF 1495 kb) [file 13287_2019_1137_MOESM5_ESM.pdf]

siNS

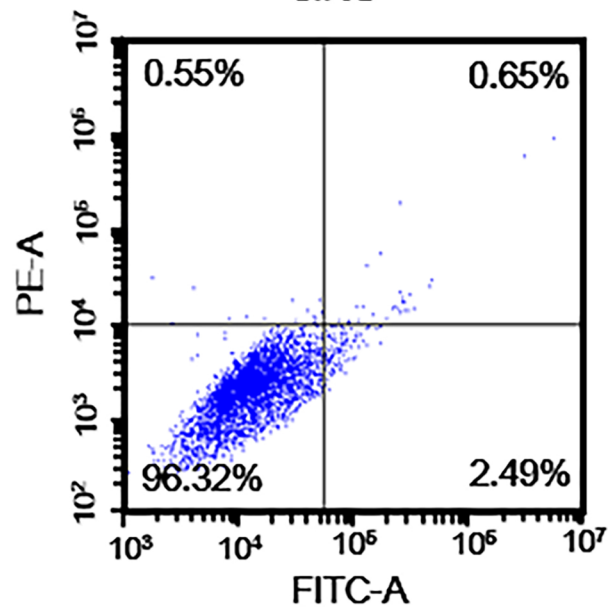

siEII3

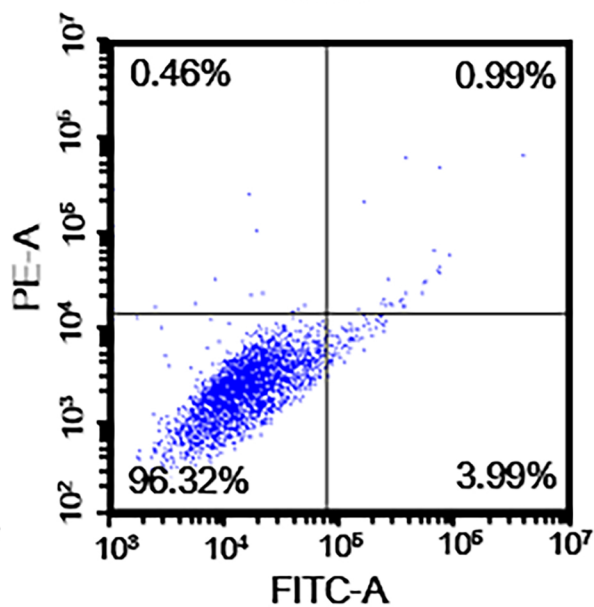

Supplementary Figure-5

Supplement: Supplementary file 6 — Figure S5. Apoptosis of ADSCs transfected with siNS or siEll3 was analyzed by Annexin V staining and flow cytometry. (PDF 1103 kb) [file 13287_2019_1137_MOESM6_ESM.pdf]
